# NSCLC depend upon YAP expression and nuclear localization after acquiring resistance to EGFR inhibitors

**DOI:** 10.18632/genesandcancer.136

**Published:** 2017-03

**Authors:** Marc McGowan, Lilach Kleinberg, Ann Rita Halvorsen, Åslaug Helland, Odd Terje Brustugun

**Affiliations:** ^1^ Department of Cancer Genetics, Radium Hospital – Oslo University Hospital, Oslo, Norway; ^2^ Department of Pathology, Oslo University Hospital, Oslo, Norway; ^3^ Institute for Clinical Medicine, Faculty of Medicine, University of Oslo, Norway; ^4^ Section of Oncology, Drammen Hospital, Vestre Viken Hospital Trust, Drammen, Norway

**Keywords:** Drug-resistance, NSCLC, YAP, EGFR, EMT

## Abstract

Yes-associated protein (YAP) is a downstream target of the Hippo pathway and has been found to be oncogenic driving many cancers into developing metastatic phenotypes leading to poor survival outcomes. This study investigated if YAP expression is associated with drug resistance in two non-small cell lung cancer (NSCLC) lines (HCC827 and H1975) generated to become resistant to the EGFR tyrosine kinase inhibitors (EGFR TKI) erlotinib, gefitinib or the T790M-specific osimertinib. We found that acquired EGFR TKI resistance was associated with YAP over-expression (osimertinib-resistant cells) or YAP amplification (erlotinib- and gefitinib-resistant cells) along with EMT phenotypic changes. YAP was localized in the nucleus, indicative of active protein. siRNA-mediated silencing of YAP resulted in re-sensitizing the drug-resistant cells to EGFR TKI compared to the negative siRNA controls (p = <0.05). These results suggest YAP is a potential mechanism of EGFR-TKI resistance in NSCLC and may presents itself as a viable therapeutic target.

## INTRODUCTION

Innon-small cell lung cancer (NSCLC), responsible for the highest death toll among cancers [1], targeted therapy has been a remarkable success. Both EGFR−, ALK−, ROS1 -directed therapies are approved, and about a fifth of all metastatic NSCLC patients may be offered such therapies with median responses of around a year [2]). However, progression due to acquired resistance is virtually inevitable, and a number of different resistance mechanisms are described [3]. In EGFR-mutated tumours treated with the first (erlotinib, gefitinib) or second (afatinib) generation EGFR tyrosine kinase inhibitors, the most frequent mechanism of resistance is a secondary mutation in exon 20 of the EGFR-gene; T790M [4]. Recently a drug targeting T790M-positive tumours, osimertinib, was approved after studies showing prolongation of progression free survival in EGFR-pre-treated and progressed patients harbouring T790M [5]. Still, a substantial fraction of tumours harbour other resistance mechanisms of which some, as AXL over-expression [6] and *MET* amplification [7, 8] are known, but others are still unknown and where no targeted therapies are available.

Yes-associated protein (YAP) has been found to both regulate the expression of Axl [9] and also drive the required phenotypic changes to cause epithelial to mesenchymal cell transformation (EMT) after binding with its transcriptional co-activator TEAD [10]. YAP expression is associated with reduced survival and relapse trends in NSCLC patients [11] which further highlights this co-transcription factor as an interesting target for drug-resistance research. But not much is known about YAP's role in drug-resistance. This study presents a new view of YAP using the HCC827 (exon 19 E746-A750 deletion) NSCLC cell line generated to become resistant to first generation TKIs and H1975 (harbouring both the T790M and L858R mutation) to osimertinib.

## RESULTS

### Drug-resistant sub-lines

**Figure 1:**
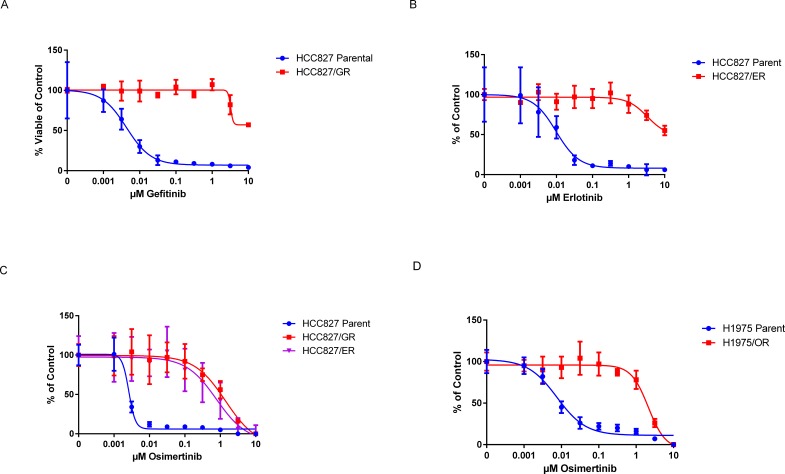
EC50 of HCC827GR, HCC827/ER, and H1975/OR sub-lines A. There was a clear difference between drug-sensitive HCC827 parental (EC_50_ = 0.004 μM) and gefitinib-resistant (GR) sub-line (EC_50_ >10 μM). B. Erlotinib-resistant HCC827/ER showed the ability to proliferate in high concentrations of drug (EC_50_ >10 μM) compared to the parental (EC_50_ = 0.001 μM). C. The HCC827/ER, GR levels of tolerance to osimertinib. The HCC827/GR sub-line showed a greater tolerance to osimertinib (EC_50_ = 1.4 μM) while the HCC827/ER had a lower tolerance (EC_50_ = 0.8 μM). It was concluded that the cells not being completely resistant to osimertinib did show the ability degree to proliferate in higher concentrations than the parental line (EC_50_ = 0.003 μM). D. The H1975/OR shows resistance to osimertinib (EC_50_ = ∼2.5 μM) compared to the H1975 parental (EC_50_ = 0.008 μM).

After proliferation was observed in the HCC827 gefitinib (GR) and erlotinib-resistant (ER) sub-lines they were isolated using cloning cylinders and expanded in individual colonies. Twenty-four sub-lines were generated and analysed for *EGFR* mutations outside of the exon 19 in frame deletion. Sequencing results showed no alterations from the HCC827 parental and drug-resistant sub-lines (data not shown). We randomly selected one erlotinib and one gefitinib sub-line for all further experiment. We determined the HCC827/ER and GR sub-lines were drug-resistant following a cell viability assay showing a shift in EC_50_values from the HCC827 parental line (Figure 1 A and B). We tested whether the third generation EGFR inhibitor osimertinib (formally AZD9291) was able to inhibit growth in the drug-resistant sub-lines compared to the parental controls using the cell viability assay. The results show a shift in EC_50_ value from the drug-resistant cells compared to the parental (Figure 1 C).

The H1975 cell line is characterized by harbouring the EGFR gatekeeper mutation T790M in exon 20 that prohibits signalling inhibition by erlotinib and gefitinib. We generated three sub-lines resistant to osimertinib (referred to as H1975/OR), whereof one sub-line was selected at random for all further experiments. The cell viability showed a shift in EC_50_ from the H1975 parental and H1975/OR – from 0.01 μM to ∼2.5 μM – (Figure 1 D). We sequenced exons 18-21 of the *EGFR* gene for additional mutations but our results showed no alterations from the parental line (data not shown).

### Resistant sub-lines promote EMT changes and Expression of YAP

**Figure 2:**
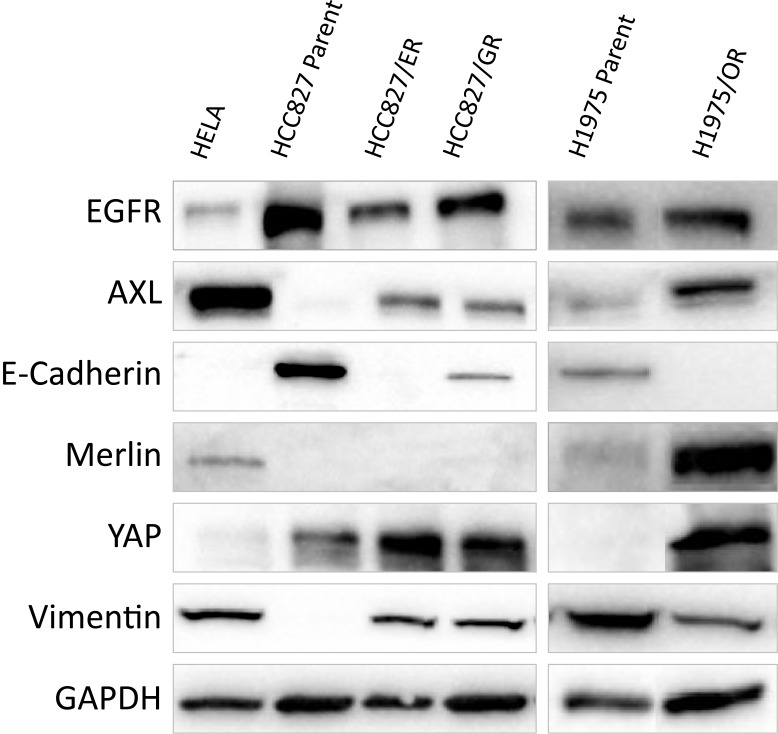
A Western Blot of HCC827 and H1975 parental and drug-resistant sub-lines characterisation EGFR fluctuated slightly from HCC827 parental and both ER and GR sub-lines. AXL expression was observed in the drug-resistant sub-lines along with EMT marker Vimentin and loss of E-Cadherin. YAP was observed in parental cells and was amplified in the drug-resistant cells. EGFR remained consistent between H1975 parental cells and H1975/OR sub-line. AXL expression was also observed in parental and appeared to increase in expression as cells acquired resistance to osimertinib. EMT marker vimentin was down-regulated but remained expressed as e-cadherin was lost in H1975/OR. Merlin expression was increased in H1975/OR sub-line with co-expression of YAP.

The HCC827/ER sub-line showed markers of EMT (vimentin expression and loss of e-cadherin) and AXL expression after acquiring resistance to erlotinib. HCC827/GR sub-lines also showed AXL and vimentin expression but still some e-cadherin expression Figure 2) compared to the HCC827/ER sub-line. The morphology of the sub-lines also differed where HCC827/ER were mesenchymal and differed from the parental, and HCC827/GR resembled their parental and did not appear to have undergone EMT. Interestingly, EGFR appeared to be down-regulated in the HCC827/ER sub-line when compared to HCC827/GR and parental line. We observed YAP over-expression after acquiring resistance to the first generation EGFR inhibitors. Using RT-qPCR confirmed amplification of YAP at the mRNA level (data not shown). We then analysed if the overexpression of YAP resulted in the increased expression of its inhibitor Merlin. We found Merlin was not expressed in any of the HCC827 parental or drug-resistant sub-lines. The H1975/OR sub-line was tested for known resistant proteins (AXL, EGFR) as well as YAP expression (Figure 2). From the Western Blot the H1975 parental cells harboured AXL which was over-expressed in the H1975/OR sub-line. We saw YAP expression in the H1975/OR sub-lines and not in the parental. We further evaluated these findings with RT-qPCR which confirmed expression differences (data not shown). We then assessed if Merlin was also expressed in these cells. Interestingly, we observed co-expression of both YAP and Merlin in the H1975/OR drug-resistant sub-line. This was not observed in the HCC827 drug-resistant cells (ER or GR) and warranted further experimentation to determine if YAP was active.

**Figure 3:**
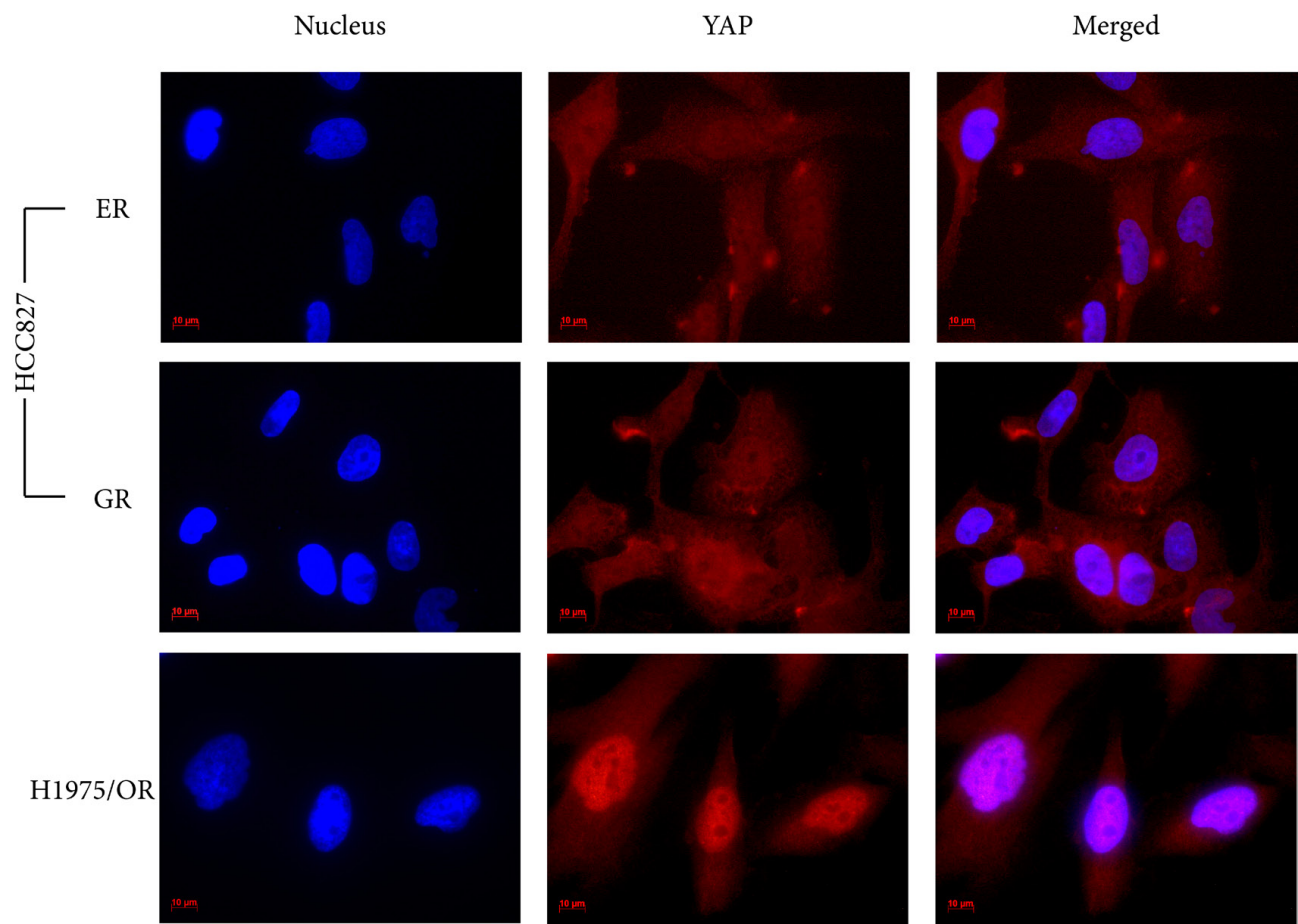
An immunocytochemistry staining showing the localization of YAP in drug-resistant sub-lines Both HCC827/ER and GR sub-lines show YAP expression distributed in the cytoplasm and nucleus. The H1975/OR sub-line shows YAP staining more focused in the nucleus than the cytoplasm. Scale bar was 10 μm.

Immunocytochemistry staining (ICC) was chosen to visualize if YAP was active (localized in the nucleus) or inactive (withheld in the cytoplasm). ICC results showed YAP was distributed in both cytoplasm and nucleus in the HCC827/ER and GR sub-lines. In the H1975/OR sub-line YAP was more predominantly localized to the nucleus than in the cytoplasm (Figure 3).

### Silencing of YAP restores drug-resistant cells sensitive to EGFR inhibitors

**Figure 4:**
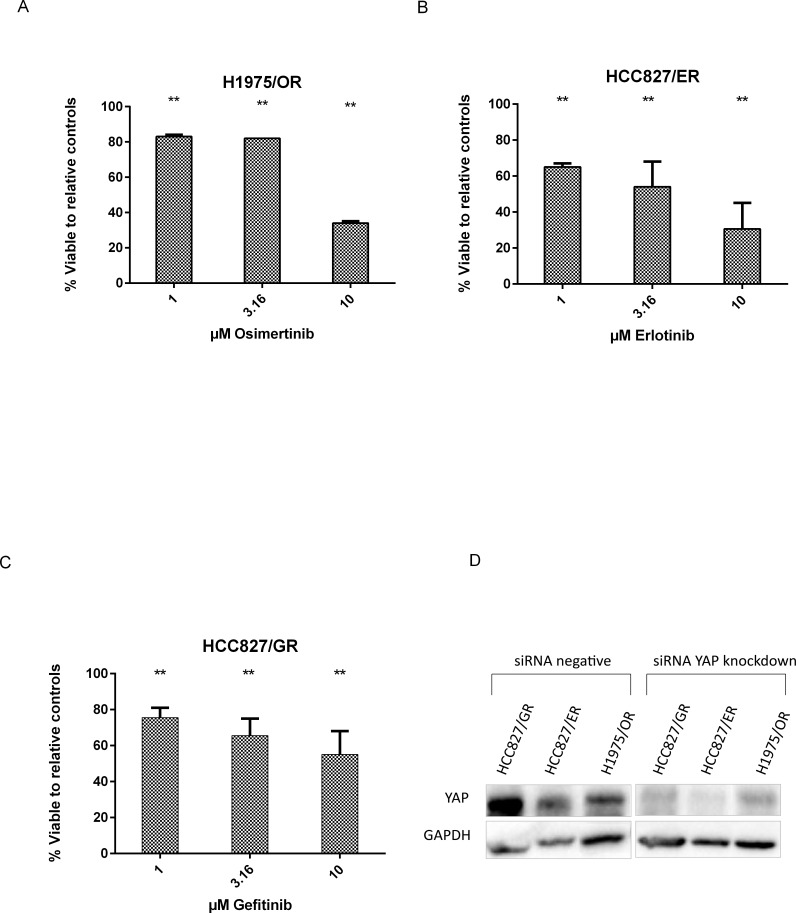
YAP siRNA knockdown allows re-sensitization to EGFR inhibitors A. H1975/OR sub-line shows reduced cell viability when re-introduced to osimertinib after YAP silencing, especially evident at doses around 10 μM. B. We observed HCC827/ER respond to erlotinib after siRNA silencing of YAP from 1 μM drug, with gradually pronounced difference from non-silenced cells up 10 μM. C. HCC827/GR sub-line showed decrease in cell viability after the introduction of drug and silencing of YAP. All siRNA knockdown wells were normalised to their negative siRNA drug counterpart and standard error was calculated based upon two repeats. D. siRNA knockdown was confirmed using Western Blot with GAPDH as a loading control. ** p = <0.05.

To further understand and evaluate YAP overexpression in relation to drug-resistance in HCC827 ER/GR and expression in H1957/OR we conducted knockdown using siRNA targeting YAP. Western Blot confirmed knockdown prior to evaluating the effects of reintroducing drug to the three drug-resistant cells (Figure 4).

H1975/OR sub-line (Figure 4 A) responded to osimertinib after silencing YAP (p = <0.05). Only 34% of the H1975/OR sub-line cells with YAP silencing siRNA were viable after normalizing to their 10 μM osimertinib negative siRNA controls. HCC827/ER sub-line Figure 4 B) responded to lower concentrations of erlotinib after silencing YAP (p = <0.05). However, HCC827/GR sub-line showed a reduced response to gefitinib compared with erlotinib-resistant cells but did show significant reduction in cell viability after YAP silencing (p = <0.05) (Figure 4 C).

## DISCUSSION

Drug resistance is a major cause of cancer treatment failure. Targeted EGFR-directed drugs show effect duration of around one year, where after resistance is inevitable. In this report we show that induction of YAP is a possible mechanism of drug resistance to EGFR tyrosine kinase inhibitors in NSCLC adenocarcinomas, and that inhibiting this co-transcription factor can re-sensitize the cells to EGFR inhibitors.

We exposed the HCC827 cell line to two first generation EGFR inhibitors, erlotinib and gefitinib, and H1975 to osimertinib (AZD9291) and found AXL, a known mechanism of EGFR-TKI-resistance [6] to be expressed in all sub-lines. This was an interesting observation as previous studies have shown that YAP may regulate AXL expression in lung adenocarcinomas [9] and in hepatocellular carcinoma by way of TEAD binding to the promoter region of the *AXL* gene [12]. Thus YAP expression and activation may be a reason for AXL induction in drug-resistant adenocarcinomas and some other cancer types; though we did not evaluate AXL expression following YAP knockdown, which would have been interesting. Future studies should investigate if YAP inhibition contributes to the reduced capability of AXL signalling which could be clinically exploited in drug-resistant cells.

We also observed a reduced band intensity of EGFR in the HCC827/ER and GR sub-lines compared to the parental (figure 2). This may indicate internalization and degradation of EGFR after erlotinib and gefitinib binding. Sakuma *et al* observed the down-regulation of EGFR after generating gefitinib-resistant HCC4006 adenocarcinoma cells – also harbouring the exon 19 E746-A750 deletion – but not in the HCC827 gefitinib-resistant sub-line [13]. This was concluded to be due to autophagocytosis and may explain why we also see this in our results after acquiring resistance to erlotinib and gefitinib. If this is also observed in the clinic it would be interesting to determine if this is also a prognostic marker of patient outcome.

We noted that the H1975/OR sub-line had a suppressed e-cadherin expression along with reduced expression of vimentin, while HCC827/ER and GR sub-lines expressed vimentin and down-regulated E-cadherin (figure 2). E-cadherin has been found to regulate the phosphorylation of YAP and promote its degradation by way of activating protein 14-3-3 localizing it to the cytoplasm and preventing the binding to TEAD [14]. Loss of the trans-cellular E-cadherin results in epithelial-mesenchymal transition and contact independent growth, migration and invasion. Vimentin induces morphological changes and increases cell motility [15]. It is conceivable that osimertinib may have off targets effecting the transcription of vimentin.

We further evaluated YAP expression and amplification in the HCC827/ER, GR and H1957/OR sublines. YAP is only active once it has sequestered to the nucleus where it binds to TEAD and begins transcription of cell survival genes and EMT capabilities [16]. We confirmed YAP expression via western blot and RT-qPCR and found YAP was distributed in both cytoplasm and cell nucleus in the HCC827/ER and GR sub-lines, but more localized to the nucleus in the H1975/OR sub-line (figure 3). We therefore suspected that YAP was both over-expressed and active in all drug-resistant sub-lines. This question, however, was limited as we only used one method of identifying YAP localization. We plan to further investigate YAP-TEAD downstream transcription targets such as amphiregulin that YAP-TEAD downstream transcription targets such as amphiregulin, which would also help confirm YAP activation. We will employ these in all further experiments evaluating YAP activity in drug-resistant NSCLC.

We then wanted to determine if YAP was involved in drug-resistance or merely acted as a biomarker. We found that siRNA-induced YAP silencing restored the negative effect of EGFR inhibitors on cell viability. Our results highlight YAP as a possible mechanism of drug resistance also for the third-generation EGFR-inhibitors, and thus confirm and extend the findings by Hsu *et al* [17] whose group showed HCC827 erlotinib-resistant and H1975 cells to become re-sensitized to erlotinib after YAP was silenced. Another group also found that inhibiting YAP re-sensitized breast cancer cells to chemotherapeutic agents demonstrating YAP's ability to orchestrate drug-resistance [18]. These, along with our results, demonstrate an emerging role of YAP in drug resistance and increase the possibility of a new therapeutic target of relapsed EGFR tyrosine kinase inhibitors. The use of statins has been studied and shown to be a potent inhibitor of YAP sequestering to the nucleus in breast cancer cell lines [19]. This study showed potential in pre-existing drugs already approved for use in the clinic which can be revaluated into a new role speeding up the use in YAP-driven cancers. We did not evaluate the expression of EMT markers and AXL post YAP silencing, which has limited our results to suggest YAP is involved with orchestrating EMT.

In conclusion, YAP may be a potential central factor in acquired EGFR-TKI resistance, and further work studying in-depth mechanisms of regulation, role in clinical prognostication and response predication as well as potential interventional approaches are warranted and ongoing. More work will need to be conducted to confirm YAP activation using the limitations from our experiments to unravel more about this co-transcription factor

## MATERIALS AND METHODS

### Cell lines and Reagents

HCC827 and H1975 were purchased from ATCC (CRL-2868 and CRL-5908 respectively) and were maintained in RPMI1640 media (R8758, Sigma) supplemented with penicillin-streptomycin (15140-122, Gibco), L-glutamine (G7513, Sigma), and 10% v/v foetal bovine serum (S1810-500, VWR) in a 95% humidified 5% CO_50_ atmosphere at 37oC. Erlotinib was purchased from LC Laboratories (E-4007), osimertinib (AZD9291) was bought from MedChem Express (HY-15772), and gefitinib was a kind gift from Solveig Pettersen at the Radium Hospital. All drugs and compounds were diluted in DMSO (D2650, Sigma) and kept at 0.1% v/v or lower in cell culture media.

HCC827 erlotinib-resistant (ER) and gefitinib-resistant (GR) sub-lines were generated by culturing early passage HCC827 parental cells stepwise from their EC_50_ to above their C_max_ values. HCC827/ER sub-lines were cultured to 3.5 μM Erlotinib and HCC827/GR sub-lines cultured to 2.5 μM Gefitinib; their C_max_ concentrations are 2.5 μM erlotinib [20] and 0.3 μM gefitinib respectively [21]. H1975/OR sub-lines took much longer to establish due to the response to osimertinib. These cells were cultured to 2.5 μM osimertinib stepwise over several months. The C_max_ value for osimertinib is between 2-3 μM [22] and thus sustaining the sub-line at 2.5 μM was deemed acceptable. Analysis of drug-resistance was determined using EC_50_ data prior to further experiments.

### YAP silencing

YAP silencing was achieved using Qiagen Flexitube siRNA (Qiagen, S104438651) and negative control siRNA (Qiagen, 1022076) diluted in Lipofectamine RNAiMAX (Thermo Fisher, 13778075) and Opti-MEM (Thermo Fisher, 31985070) used as described in the manufacturers specifications. Protein knockdown was assessed using Western Blot.

### EC_50_ cell viability assay

Parental cells were assessed for sensitivity to TKI along with their respective TKI-resistant sub-lines. Cells were seeded at a concentration of 5 × 10^3^ into 96 x well plates with their respective media and allowed to adhere overnight. After 24 hours the media was removed and replaced with media containing varying concentrations of erlotinib, gefitinib or osimertinib and DMSO only for control wells at half-log dilutions. DMSO was kept consistent at 0.1% v/v for all treatment groups and controls. A resazurin metabolism assay (R7017, Sigma) was performed by making a 1/20 dilution of 2.3 mg/ml resazurin stock in sterile Dulbecco's phosphate buffered saline (DPBS) (14190094, Thermo Fisher) and using a 1/10 dilution directly in the wells after 120 hours drug incubation. Resazurin sodium salt was allowed 4 hours incubation at 37°C in a 95% humidified, 5% CO_2_ atmosphere. Plates were assessed for active viable cells using fluorescence imager BioTek Synergy 2 with software Gen5 with filters for excitation at 544 nm and emission at 590 nm. The EC_50_ curve was created using GraphPad v.6 software by normalising the average treatment wells to the DMSO controls. Data was presented as percent of controls.

For siRNA-erlotinib/gefitinib/osimertinib combination, we loaded each well of a 96 well plate with 5 × 10^3^ cells per well and left them to adhere overnight in media with drug. The following day media was changed to media with siRNA YAP or negative control siRNA and allowed to incubate for 48 hours without drug. After 48 hours we added drug dilutions directly onto the siRNA loaded cells and incubated for a further 72 hours before assessing viable cells using the resazurin assay. We normalized each siRNA drug/compound group to the negative groups, for example 10 μM erlotinib YAP siRNA was normalized to 10 μM negative siRNA control. Results were presented as percentages of the negative siRNA in a bar graph with ± SEM and statistical differences between controls and YAP knockdown were conducted using a two-tailed, unpaired and unequal variance student's T-Test and presented on the graphs.

### Immunocytochemistry of YAP

Cells were trypsinized and seeded at a concentration of 10 × 10^4^ onto frosted-coated glass slides and left to adhere overnight at 37°C in a 95% humidified 5% CO_2_ incubator. Once adhered, slides were rinsed twice with sterile D-PBS and treated with 0.3% v/v Triton-X100 (T8787, Sigma) for five minutes to permeabilize the cells. Slides were washed three more times before blocking with 5% Goat serum (16210, Thermo Fisher) for 30 minutes before incubating with either primary YAP antibody (PA1-46189, Thermo Fisher) or 5% Goat serum (staining control) for 1 hour at room temperature. Slides were washed three times and treated with Goat Anti-Rabbit IgG Alexa Fluor^®^ 594 conjugated secondary antibody (A-11037, Thermo Fisher) for 60 minutes in the dark at room temperature. Slides were washed another three times and stained with ProlLong^®^ Gold Antifade Mountant with DAPI nucleus dye (P36931, Thermo Fisher) for 10 minutes. Slides were mounted and used immediately for imaging and then stored at-20°C in the dark.

### Western Blot

Cells were harvested for protein by using Ripa lyses buffer (89901, Thermo Scientific) with 50 mM sodium orthovanadate (S6508, Sigma), 50 mM Pefabloc (1.24839.0500, Merck), 50 mM sodium fluoride (S6508, Sigma), PhosSTOP (04906845001, Roche) and Protease inhibitor cocktail (05892970001, Roche). Protein concentration was measure using the Pierce BCA kit (23227, Thermo Scientific) at the manufacturer's specifications. A concentration of 15 μg protein lysate was boiled with Laemmli sample buffer (161-0747, Bio-Rad) with 100 mM DTT (A3668, AppliChem) for five minutes at 95°C then loaded into a 12% SDS mini-PROTEAN^®^ TGX^TM^ gel (456-1046, Bio-Rad) and ran for 60 minutes at 200 v. Gels were transferred to a Midi Format nitrocellulose membrane (1704159, Bio-Rad) using a Turbo blot transfer module form Bio-Rad. The membranes were blocked for 60 minutes in (1706404, Bio-Rad) 0.1% Tween 20 (1610781, Bio-Rad) diluted in Tris buffered saline (1706435, Bio-Rad) containing 5% v/v fat-free milk. Membranes were washed for five minutes three times in 0.1% TTBS before sectioning and incubating overnight at 4°C rotating in primary antibodies: Axl (8661, Cell Signalling), EGFR (4267, Cell Signalling), Merlin (PA5-35316, Thermo Scientific), YAP (PA5-13504, Thermo Scientific), E-Cadherin (ab1416, Abcam), Vimentin (ab92547, Abcam) and GAPDH for loading control (MAB374, Millipore). Membranes were washed three times for five minutes in 0.1% TTBS before incubating with secondary antibodies for 60 minutes at room temperature in either peroxidase-conjugated Goat anti-Mouse (31431, Thermo Scientific) or Goat anti-Rabbit (31466, Thermo Scientific). Membranes were washed another three times for five minutes in 0.1% TTBS and developed using SuperSignal^®^ (34095, Thermo Scientific) and being viewed using the Bio-Rad Chemi Doc ™ MP imager running Image Lb v4.1 software.

## Abbreviations

EGFR: Epidermal growth factor receptor
EMT: Epithelial to mesenchymal transition
YAP: Yes-associated protein
